# Delayed enterocutaneous fistula after 17 years of gunshot wound injury: a case report

**DOI:** 10.1097/MS9.0000000000000886

**Published:** 2023-06-05

**Authors:** Nuradin M. Nur, Najib M. Salad, Abdullahi A. Ahmed

**Affiliations:** a General Surgery Department; b Thoracic Surgery Department, Mogadishu Somali Turkey, Recep Tayyip Erdogan Training and Research Hospital, Somalia

**Keywords:** a case report, colocolic anastomosis, enterocutaneous fistula, gunshot

## Abstract

Enterocutaneous fistula (ECF) is a common complication of abdominal surgery. Postoperatively, it may be the result of a small intestinal injury or an anastomosis leak, and it typically manifests days or weeks following surgery.

**Case presentation:**

A 56-year-old man with a history of a gunshot wound injury in the abdomen with colon injury managed for colocolic anastomosis 17 years earlier presented to our hospital’s general surgery clinic with the complaint of drainage at the scar area. It was discovered that he had an ECF. The patient underwent a laparotomy. Fistula tract excision and segmental colon resection with colocolic anastomosis were done.

**Clinical discussion:**

ECF formation after an extraordinarily long latency due to an anastomotic leak, which has not been previously documented in the literature, is a distinctive aspect of the case. As a result, surgeons should consider ECF in their differentials for patients with a history of abdominal operations.

**Conclusion:**

Surgical management is the definitive treatment of ECF after proper resuscitation and stabilization of the patient’s condition. Preoperative imaging is crucial for determining the anatomy of the fistula and any associated intra-abdominal pathology.

## Introduction

Highlights

- Delayed enterocutaneous fistula after 17 years of colon anastomosis is extremely rare, as in our case.
- In spite of a sepsis-free interval of conservative management surgical treatment has a good chance of being successful for patients who have a healed wound associated with a fistula opening.
- Although enterocutaneous fistula presents early postoperatively, it can also present late, as in our case, and always carries higher morbidity and mortality, so careful consideration of the patient’s history with a definitive diagnosis reduces such complications.

An enterocutaneous fistula (ECF) is an inappropriate connection between the gastrointestinal (GI) tract and the skin or a wound^1^. The ECF is a well-known intra-abdominal surgical complication. While iatrogenic causes account for 75–85% of ECFs, the remaining 15–25% are caused by spontaneous processes^2^. Following both laparoscopic and open surgeries, it may develop as a result of small intestinal damage or an anastomotic leak^3^.

In this case report, we describe a rare occurrence of colocutaneous fistula that developed 17 years after colocolic anastomosis in trauma and was treated with a definitive surgical procedure.

## Case report

A 56-year-old man arrived at our outpatient clinic complaining of recent mild abdominal pain and barely detectable feculent discharge from his left lower quadrant of the abdomen. He had a history of gunshot wound injuries 17 years ago, including a left colon injury in which he underwent colocolic anastomosis.

Clinically, the patient’s vital signs were normal, and a tiny scar with a feculant discharge was visible in the left lower quadrant of the abdomen. The skin around it appeared to be normal.

After normal laboratory tests, radiological imaging was done.

**Figure 1:**
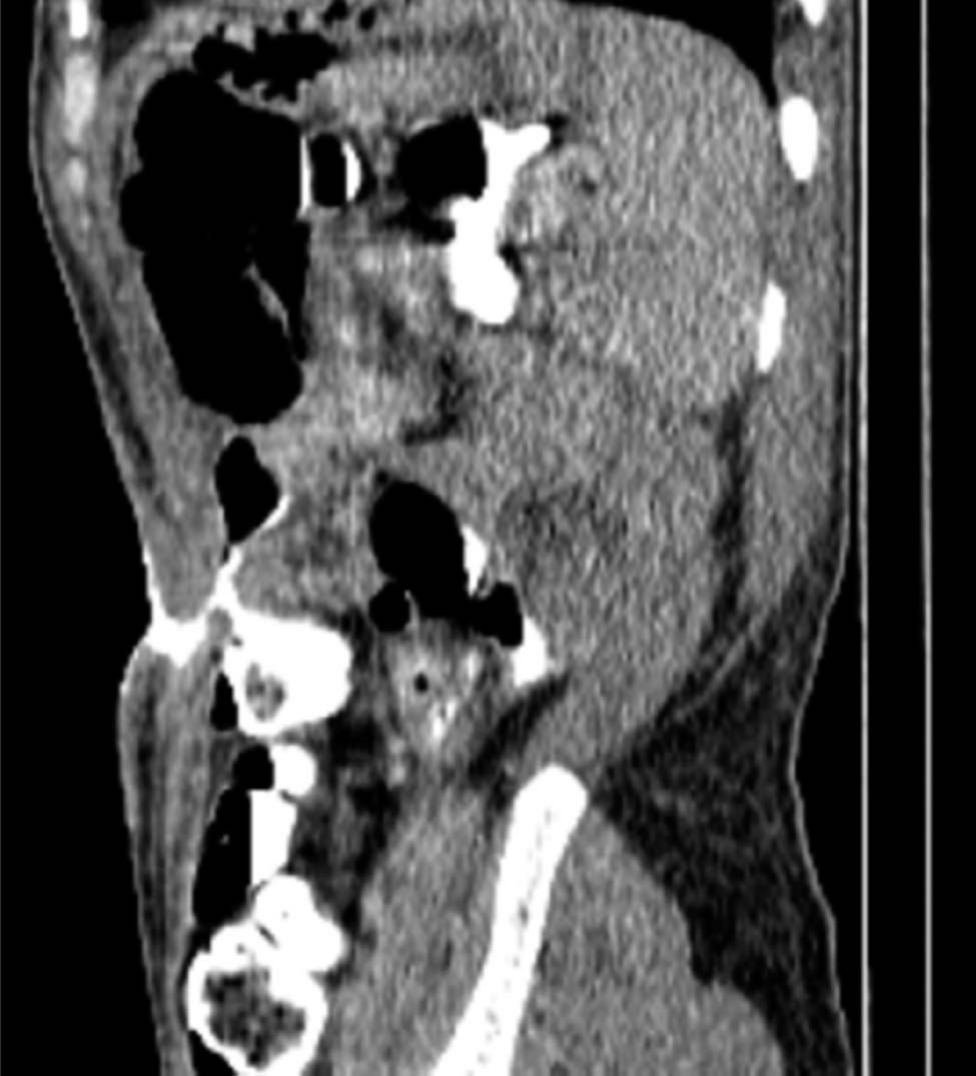
Oral contrast computed tomography showing the fistula tract.

A computed tomography (CT) abdomen with IV and oral contrast was performed in which there is a fistulous track in the left lower quadrant extending intra-abdominally to reach the descending colon, hence identifying a colocutaneous fistula (Fig. 1).

**Figure 2:**
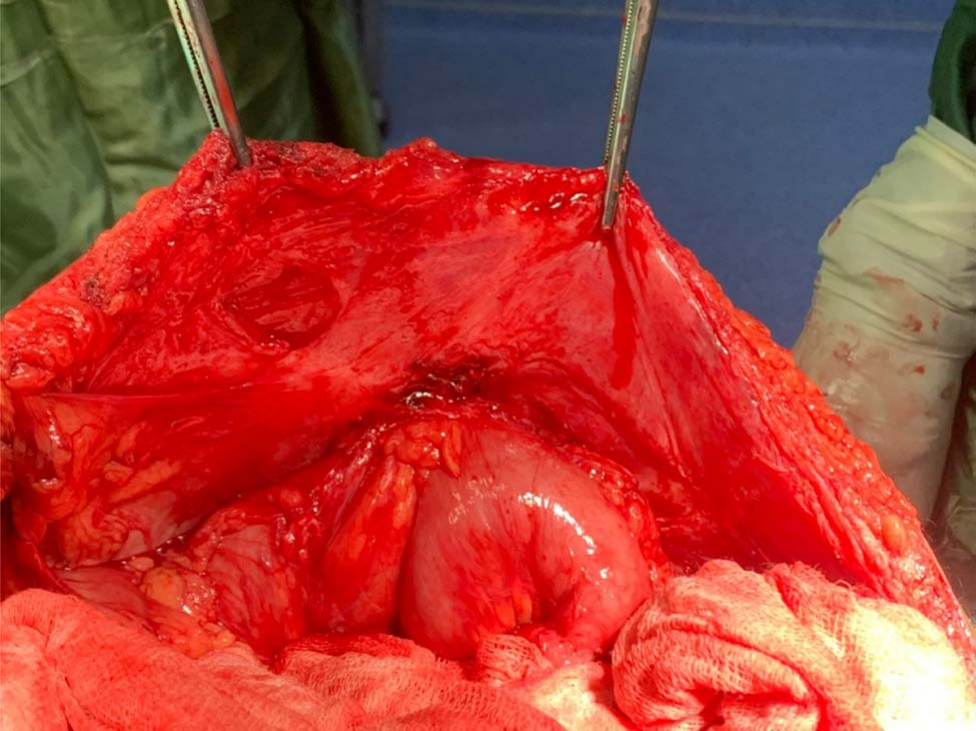
Loops of small bowel adherent to the colonic fistula.

**Figure 3:**
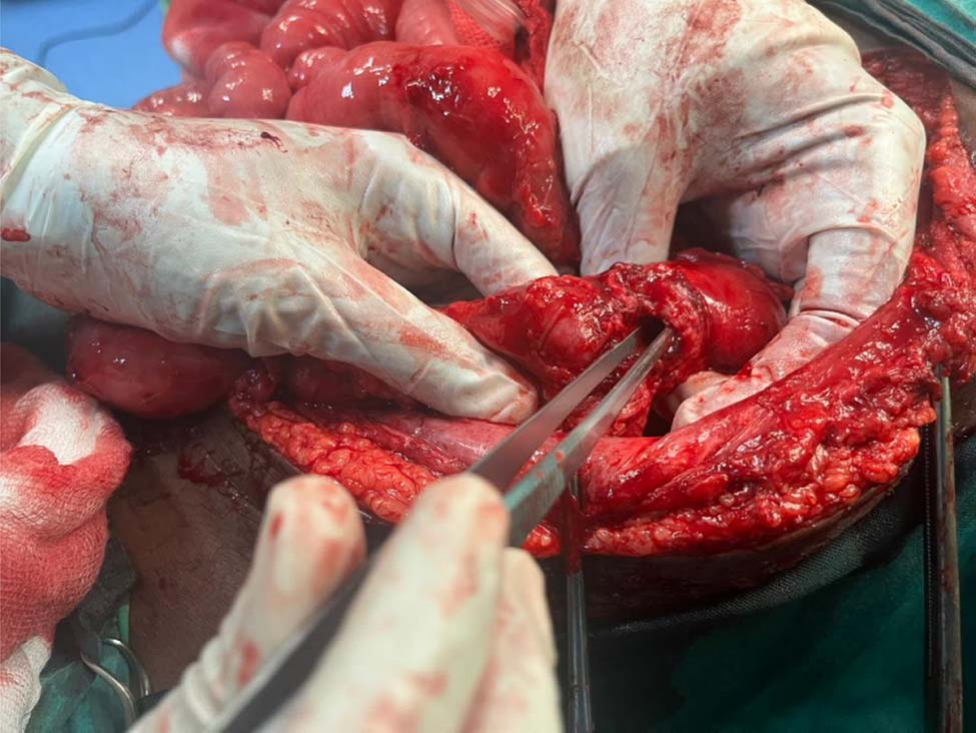
Opening of the fistula in the descending colon.

After completing the preoperative workup, an exploratory laparotomy was done, and the abdomen was explored. A fistulous tract was seen communicating with the descending colon. There were also loops of small bowel adhered to the colon (Fig. 2); adhesiolysis was done, and there was a 2 cm defect in the colon (Fig. 3). Resection and anastomosis were done with a stapler.

Histopathology was performed on the resected specimen and showed congestion, ulceration, chronic inflammation, and a fistulous tract with colon tissue.

The postoperative phase was uncomplicated, and on the sixth postoperative day, the patient was discharged and given instructions for routine follow-up.

## Discussion

After abdominal surgery, ECFs are still a terrible consequence that can be difficult to treat. In the postoperative phase, between days 5 and 10, the typical presentation is observed^4^. We are reporting an unusually delayed presentation of ECF after 17 years of colon anastomosis in trauma.

On the basis of the patient’s medical history, general nonspecific constitutional symptoms and other particular symptoms, such as feculent or bilious wound discharge, diarrhea, and GI hemorrhage, are discernible. Imaging with GI contrast that traverses the fistula (fistulogram) typically confirms the diagnosis. Nevertheless, a CT scan is frequently the main diagnostic modality of choice, is highly specific in defining the anatomy of the fistulous tract, and frequently excludes the existence of an abdominopelvic abscess, as intra-abdominal abscesses are associated with fistula in 44% of patients^5^. In this case, the appearance of a feculant leak and a CT abdomen with IV and oral contrast suggested the diagnosis.

Patients with conventional fistulas and no unfavorable variables should undergo surgical intervention if they are stable^6^, have no source of sepsis, and can tolerate the resectional technique required for fistula closure^7^. It is also essential that the process be technically viable without posing a very high risk of damage to the intestine or other important structures.

Surgical treatment has a good chance of being successful for patients who have a healed wound associated with a fistula opening^6^. The patient underwent definitive surgical management, and a favorable outcome was observed during follow-up.

This activity has been documented in line with the Surgical CAse REport (SCARE) 2020 criteria^8^.

## Conclusion

This case report highlights the incidence of ECF following colocolic anastomosis, which manifests 17 years after surgery. Although preventing this complication is extremely difficult, it can be lessened with good anastomotic technique and the management of underlying local or systemic disease. As a result, the surgical team performing colon surgery must exercise extreme caution for the early detection of ECFs because they can present themselves either early or late, as in this case. In order to avoid morbidity and complications, definitive surgical intervention is the primary course of treatment.

## Ethics approval and consent to participate

Ethical approval was waived by the ethical committee of Mogadishu Somali Turkey, Recep Tayyip Erdogan Training and Research Hospital. Written informed consent was obtained from the patient for participation.

## Consent for publication

Written informed consent was obtained from the patient for the publication of this case report and accompanying images. A copy of the written consent is available for review by the Editor-in-Chief of this journal on request.

## Sources of funding

No funding was received.

## Author contribution / Credit author statement

N.M.N.: conceptualization, data curation, visualization, investigation writing, original draft preparation; N.M.S.: writing, reviewing, and editing; A.A.A.: reviewed and revised the manuscript for intellectual content critically.

## Conflicts of interest disclosure

This manuscript has not been submitted to, nor is it under review at, another journal or other publishing venue. The authors have no affiliation with any organization with a direct or indirect financial interest in the subject matter discussed in the manuscript.

## Research registration unique identifying number (UIN)

Name of the registry: NA.
Unique Identifying number or registration ID: NA.
Hyperlink to your specific registration (must be publicly accessible and will be checked): NA.

## Guarantor

Nuradin Mohamed Nur.

## Availability of data and materials:

The data that support the findings of this study are available in Mogadishu Somali Turkey, Recep Tayyip Erdogan Training and Research Hospital information system. Data are; however; allowed to the authors upon reasonable request and with the permission of the education and research committee.

## Provenance and peer review

Not commissioned, externally peer-reviewed.

## Acknowledgements

The authors acknowledge the education and research committees of Mogadishu, Somalia, Turkey, Recep Tayyip Erdogan Training and Research Hospital. The authors acknowledge S.A, who allowed us to use her clinical information, reports, and images for this case report.
